# Oral health status and caries trend among 12-year old Palestine refugee students: results from the UNRWA’s oral health surveys 2011 and 2016

**DOI:** 10.1186/s12903-019-0844-z

**Published:** 2019-07-18

**Authors:** Lilia Biscaglia, Patrizia di Caccamo, Irene Terrenato, Maria Antonietta Arrica, Akihiro Seita, Guglielmo Campus, UNRWA group, Ali Khader, Majed Hababeh, Amjad Issa, Hanan Diab, Imad Burbara, Zaki ElSaleh, Ayoub Ayoub, Mohammad Salameh, Mohammad Hadi Saleh, Amjad Matooq, Abdalla Odattalla, Asa’ad Na’jeh, Akiko Kitamura, Julia McCahey, Gloria Paolucci

**Affiliations:** 1 COI Cooperazione Odontoiatrica Internazionale NGO, Turin, Italy; 2 Biostatistics and Bioinformatic Unit- Scientific Direction, IRCCS - Regina Elena National Cancer Institute, Rome, Italy; 3 Department of Surgery, Medicine and Experimental Sciences, School of Dentistry, University of Sassari, Sassari, Italy; 4 Department of Health, UNRWA Headquarters Amman, Amman, Jordan; 5 Klinik für Zahnerhaltung, Präventiv- und Kinderzahnmedizin Zahnmedizinische Kliniken (ZMK), University of Bern, Freiburgstrasse 7, Bern, Switzerland

**Keywords:** Oral health, Dental health surveys, Dental caries, Health status indicators, Risk factors

## Abstract

**Background:**

In 2016 the United Nation Relief and Work Agency for Palestine refugees in the Near East (UNRWA) commissioned a survey on oral health among 12-year-old students at UNRWA schools in five fields of operation (Jordan, Lebanon, Syria, Gaza Strip and West Bank), following World Health Organization guidelines. The survey aimed to determine the prevalence of dental caries and periodontal diseases among Palestine students attending UNRWA schools and how this has changed over time.

**Methods:**

A two-stage stratified cluster sample design was used. For each Field of operation, the sample size was calculated based on 95% confidence level, 80% power and margin of error of 4%. Clinical examination was carried out by trained Field Oral Health services Officers (FOHSOs) from the 5 fields. Teeth presence and condition, gingival bleeding and calculus and the presence of dental sealants in occlusal surfaces of permanent molars were recorded. Behavior information of students/parents were collected using a questionnaire that was self-completed by the child/parent under supervision. Results were compared with those from a previous survey carried out in 2011 with the same methodology.

**Results:**

In the two surveys the distributions of students who had caries experience in their permanent teeth were similar (73.1% in 2011 vs 72.8% in 2016, *p* = 0.83). In 2016 a significant increase of missing teeth (*p* < 0.01) and sealants (*p* < 0.01) was observed. Both surveys have identified behavioral determinants for dental caries, particularly dietary habits such as soft drinks consumption. Gingival health also showed statistical differences among the fields.

**Conclusions:**

The prevalence of caries experience was very high in all fields and, with regard to main oral health indices, no trend of improvement was observed through 2011 and 2016. Surveys’ results advocates the need of a large-scale integrated preventive approach toward oral health and the emerging growth of Noncommunicable Diseases (NCDs), in line with the WHO recommendations.

## Background

The burden of oral diseases is growing in several developing countries due to the nutrition transition and to inadequate exposure to fluorides [1–3]. In resource-limited settings the double-burden of malnutrition, a co-existence of undernutrition and diets high in fats and sweeteners [4], is becoming an increasing concern which also affects oral health [5].

Oral disease burden is, in particular, a major health problem among Palestine refugees [6]. The United Nation Relief and Work Agency for Palestine refugees in the Near East (UNRWA) was established in 1949, and it provides education, health care, social services, relief, protection and other essential services to Palestine refugees in the Middle East [7]. To date, UNRWA services are available to almost 5.4 million of registered Palestine refugees present in area of operations (Jordan, Lebanon, Syria, Gaza Strip and West Bank), who live both inside and outside of refugee camps [6]. Oral health services are provided by UNRWA through 106 dental clinics integrated within the Agency’s primary health care facilities and 9 mobile dental teams [8]. In the last decade UNRWA has strengthened population-based preventive interventions aimed to effectively tackle the oral health disease burden [9]. Under the UNRWA School Health Program, which reaches more than 500,000 students annually, oral health screening for students in the first, seventh and ninth grades is provided. Screening is coupled with other dental caries prevention activities such as pit and fissure sealant for first- or second-grade students with a first erupted molar, fluoride mouth rinsing and tooth-brushing campaigns [6]. In May 2008, UNRWA commissioned an evaluation of its oral health services to COI (Cooperazione Odontoiatrica Internazionale) NGO. Among other recommendations, the evaluation highlighted the need for standardized epidemiological survey procedures according to the WHO guidelines across fields. Support and guidance from COI continued over the years, with the involvement of the WHO Collaborating Center (WHOCC) of Milan and lead to this oral health survey conducted in the academic school year 2010/2011. 

In 2016 UNRWA commissioned a second survey on oral health among 12-year-old Palestine students according to the World Health Organization (WHO) guidelines, with the aim to monitoring disease patterns and trends over time and measuring the progress, impact and efficacy of oral health preventive interventions [10, 11].

The main aim of this paper is to determine the prevalence of dental caries and periodontal diseases among Palestine students attending UNRWA schools and how this has changed over time. A secondary aim is to describe the oral health related behavior of the study population and again how this has changed over time.

## Methods

### Study design

The cross-sectional oral health surveys were conducted in the five fields of operations of the Agency: Jordan, Lebanon, Syria, Gaza Strip and West Bank. The surveys were carried out between March and June 2011 and February and May 2016, respectively.

The population object of the survey included all male and female 7th grade students enrolled in UNRWA schools.

The surveys design followed Helsinki declaration and were approved by UNRWA authorities. An information leaflet, explaining the aim of the study and requesting their child’s participation with a signed consent, was given to parents or guardians. Only children with parents’ signed consent were called for examination.

A multistage cluster sampling was performed, considering the five fields as strata; in the second stage the list of classes and schools were compiled into a list and then the secondary schools were chosen at cluster level with proportional random selection of participants.

**Fig. 1:**
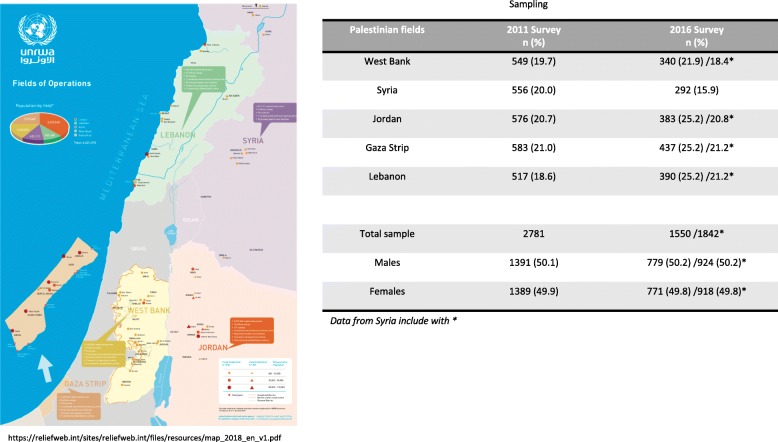
Demographic characteristics of the study participants. The UNRWA oral health surveys 2011–2016

For each Field of operation the sample size was calculated based on 95% confidence level, 80% power and margin of error of 4%. A sum of the estimated sample size in the five fields was calculated to extrapolate a wide sample representative of the overall population (Fig. 1).

### Data collection

Oral health status was assessed by clinical examination according to the method and criteria recommended by the WHO [11]. Clinical examination was carried out by trained Palestine Field Oral Health services Officers (FOHSOs) from the 5 fields. In each survey 11 FOHSOs attended a two-day training session under the supervision of a team of trainers from the WHO Collaborating Centre (WHOCC) of Milan and a non-governmental organization (NGO) called COI (Cooperazione Odontoiatrica Internazionale). Eight of the 11 FOHSO were involved in both the 2011 and 2016 surveys. Inter-examiner reliability was evaluated through fixed effect analysis of variance [12, 13]. At the end of the training courses, 95% inter-examiner agreement was achieved.

According with the WHO methodology [11], visual tactile examinations were conducted with the aid of a mirror, a standardized WHO probe and a light source. Examiners also used gloves, masks and gauzes as disposables. Clinical examination included: teeth presence and condition (coronal caries and root caries), gingival bleeding and calculus and the presence of dental sealants in occlusal surfaces of permanent molars.

Information on the behavior of students and on parents was collected using a questionnaire that was self-completed by the child/parent under supervision. The questionnaire was identical in 2011 and 2016. The questionnaire was previously standardized for a Italian National survey on Oral Health in children [14]. The questionnaire included 19 questions, grouped into four domains: socioeconomic status, dietary habits, oral hygiene habits, and dental attendance patterns of child and parents. Furthermore, demographic characteristics such as age, gender, field and place of residence (inside/outside camps) were recorded. All data were recorded on standardized forms and entered into a spreadsheet for analysis. Random checking was undertaken to verify the accuracy of data entry.

### Data analysis

Both descriptive and analytic approaches were used for data analysis. In line with the WHO methodology [11], decayed, missing and filled teeth or surfaces (DMFT/S) indices were used to assess dental caries [15]. These indices indicate the number of teeth/surfaces that were affected by caries, filled or missing as a result of caries. Decayed teeth/ surfaces (DT/S), missing teeth/surfaces (MT/S), filled teeth/surfaces (FT/S) and mean DMFT/S scores were calculated. The percentage of students whose DMFT/S was greater than 0 was used to evaluate the prevalence of tooth decay (caries experience, which includes untreated and restored lesions), whilst the percentage of participants whose DT/S was greater than 0 was calculated to evaluate the prevalence of untreated tooth decay. The mean of DMFS for the one-third of the study group with the highest caries scores (DMFS SIC - Significant Caries index) was calculated [16–18]. The percentage of DS or FS among students with at least one DMFS was calculated. The Community Periodontal Index (CPI) was used to record the periodontal status [11]. With reference to dental sealants, students were coded as having sealants when one or more permanent teeth were sealed.

The Pearson’s Chi-square, the Mann-Whitney and the Kruskal-Wallis non parametric tests were performed, when appropriate, both on the total sample and sorting data by Field and gender in order to identify any relevant association.

Relationships between dental caries prevalence and the factors collected during questionnaire administration: gender, field, sugary product consumption, meal frequency, oral hygiene habits (tooth brushing frequency and fluoridated dentifrice), mother’s education, father’s education, dietary habits (meal frequency, sugar intake frequency, sugar intake between meals, soft drinks consumption during meals, soft drinks consumption between meals), tooth brushing habit and the four variables exploring dental attendance (child, mother, and father attendance) were assessed using univariate and multivariate logistic regression models, following a forward selection method. These analyses were conducted separately for each survey. The dependent variable, DMFS, was dichotomized as DMFS = 0 versus DMFS > 0 and then was adjusted for the variables statistically significant in the univariate models. A *p*-value less than 0.05 was considered statistically significant.

In 2016, in Syria a lower than expected response rate was achieved. Furthermore, after performing sensitivity analyses on the main findings by Field, data from Syria were considered not representative for the studied population. For this reason, data from Syria from both the surveys were excluded from the analysis.

The data were analyzed using SPSS software (SPSS version 21.0, SPSS Inc. Chicago, Illinois USA).

## Results

### Demographic characteristics of the study participants

Table 1 shows the demographic information of the students involved in the 2011 and 2016 surveys. The sample sizes were 2,781 in 2011 and 1,550 in 2016, the reason for smaller sample size enrolled due to the situation in Syria as described above. Girls made up 49.9 and 49.8% of the students in 2011 and 2016, respectively.

**Table 1:** Oral Health indices. The UNRWA oral health surveys 2011 and 2016

	Survey 2011 *N* = 2781	Survey 2016^a^ *N* = 1550	*p*-value*
	N	N	
Present Teeth			< 0.01**
Median (min;max)	28 (15–28)	26 (12–28)	
DS			
Median (min;max)	2 (0–40)	2 (0–32)	
Mean (Standard Deviation, SD)	3.20 (4.23)	3.29 (3.99)	0.08**
= 0 (%)	889 (32.0)	474 (30.6)	0.35
> 0 (%)	1892 (68.0)	1076 (69.4)	
MS			
Median (min;max)	0 (0–15)	0 (0–10)	
Mean (SD)	0.14 (0.90)	0.22 (1.12)	0.01**
= 0 (%)	2708 (97.4)	1487 (95.9)	0.01
> 0 (%)	73 (2.6)	63 (4.1)	
FS			
Median (min;max)	0 (0–18)	0 (0.12)	
Mean (SD)	0.58 (1.55)	0.48 (1.34)	0.02**
= 0 (%)	2189 (78.7)	1267 (81.7)	0.02
> 0 (%)	592 (21.3)	283 (18.3)	
FT			
Median (min-max)	Not collected	(0–11)	
Mean (SD)		0.39 (0.98)	
= 0 (%)		1267 (81.7)	
> 0 (%)		283 (18.3)	
DMFS			
Median (min;max)	2 (0–40)	3 (0–32)	
Mean (SD)	3.93 (4.71)	3.99 (4.59)	0.45**
= 0 (%)	747 (26.9)	421 (27.2)	0.83
> 0 (%)	2034 (73.1)	1129 (72.8)	
DMFT			
Median (min-max)	Not collected	2 (0–21)	
Mean (SD)		2.52 (2.51)	
= 0 (%)		421 (27.2)	
> 0 (%)		1129 (72.8)	
SEALANTS			
Median (min;max)	0 (0–10)	0 (0–9)	
Mean (SD)	0.20 (0.89)	0.38 (1.32)	< 0.01**
= 0 (%)	2603 (93.6)	1398 (90.2)	< 0.01
> 0 (%)	178 (6.4)	152 (9.8)	

*Pearson’s Chi Square test otherwise specified; **Mann-Whitney non parametric test; ^a^ data from Syria excluded

### Oral health indices

In Table 1 oral health indices from the two surveys are compared. The overall mean caries experience expressed as DMFS was 3.93 in 2011 (Standard Deviation, SD 4.71) and 3.99 in 2016 (SD 4.59). No statistically significant differences were found (*p* = 0.49). Overall, the median value of present teeth was 28 in 2011 and 26 in 2016 (*p* < 0.01). When comparing the oral health indices from the two surveys, we observed similar distributions for students who had untreated tooth caries lesions (DS > 0, 68.0% in 2011 vs 69.4% in 2016, *p* = 0.35) and caries experience in their permanent teeth (DMFS> 0, 73.1% in 2011 vs 72.8% in 2016, *p* = 0.83). On the contrary, in 2016 we observed a significant increase of students with missing teeth (MS > 0, 2.6% vs 4.1%, *p* = < 0.01) and with sealants (6.4% vs 9.8%, *p* < 0.01), along with a significant decrease in the share of students with filled surface in permanent teeth (FS > 0, 21.3% vs 18.3%, *p* = 0.01).

In 2011, the caries prevalence (DMFS> 0) (Table 2) was statistically significant different among the five fields (*p* < 0.01), with the highest value observed in the West Bank (85.1%) and the lowest in Lebanon (68.5%). Overall, all the measurements showed a statistically significant difference among fields. As in 2016, the highest value observed in the West Bank (79.7%), while unlike the results of 2011, the lowest value was in Jordan (68.4%) (Table 3).

**Table 2:** Oral health information. The UNRWA oral health survey by field (2011)

	West Bank	Jordan	Gaza Strip	Lebanon	Syria	*p*-value
	N (%)^a^	N (%)	N (%)	N (%)	N (%)	
Present Teeth						< 0.01°
Median (min;max)	28 (15–28)	28 (20–28)	28 (20–28)	28 (20–28)	28 (18–28)	
DS						< 0.01^#^
0	92 (16.8)	200 (34.7)	207 (35.5)	212 (41.0)	178 (32.0)	
> 0	457 (83.2)	376 (65.3)	376 (64.5)	305 (59.0)	378 (68.0)	
MS						< 0.01^#^
0	518 (94.4)	555 (96.4)	574 (98.5)	515 (99.6)	546 (98.2)	
> 0	31 (5.6)	21 (3.6)	9 (1.5)	2 (0.4)	10 (1.8)	
FS						< 0.01^#^
0	447 (81.4)	414 (71.9)	488 (83.7)	394 (76.2)	446 (80.2)	
> 0	102 (18.6)	162 (28.1)	95 (16.3)	123 (23.8)	110 (19.8)	
DMFS						< 0.01^#^
0	82 (14.9)	163 (28.3)	182 (31.2)	163 (31.5)	157 (28.2)	
> 0	467 (85.1)	413 (71.7)	401 (68.8)	354 (68.5)	399 (71.8)	
SEALANTS						< 0.01^#^
0	547 (99.6)	541 (93.9)	578 (99.1)	407 (78.7)	530 (95.3)	
> 0	2 (0.4)	35 (6.1)	5 (0.9)	110 (21.3)	26 (4.7)	

^a^ Otherwise specified; °Kruskal-Wallis non parametric test; ^#^ Pearson’s Chi-square test

**Table 3:** Oral health information. The UNRWA oral health survey by field (2016)

	West Bank	Jordan	Gaza Strip	Lebanon	*p*-value
	N (%)^a^	N (%)	N (%)	N (%)	
Present Teeth					< 0.01°
Median (min-max)	27 (12–27)	26 (12–27)	25 (12–27)	26 (12–27)	
DS					< 0.01^#^
= 0	79 (23.2)	138 (36.0)	135 (30.9)	122 (31.3)	
> 0	26 (76.8)	245 (64.0)	302 (69.1)	268 (68.7)	
MS					< 0.01^#^
= 0	324 (95.3)	383 (100)	421 (96.3)	359 (92.1)	
> 0	16 (4.7)	0 (0)	16 (3.7)	31 (7.9)	< 0.01^#^
FS					
= 0	278 (91.8)	287 (74.9)	374 (85.6)	328 (84.1)	
> 0	62 (18.2)	96 (25.1)	63 (14.4)	62 (15.9)	
FT					< 0.01^#^
= 0	278 (91.8)	287 (74.9)	374 (85.6)	328 (84.1)	
> 0	62 (18.2)	96 (25.1)	63 (14.4)	62 (15.9)	
DMFS					< 0.01^#^
= 0	69 (20.3)	121 (31.6)	128 (29.3)	103 (26.4)	
> 0	271 (79.7)	262 (68.4)	309 (70.7)	287 (73.6)	
DMFT					< 0.01^#^
= 0	69 (20.3)	121 (31.6)	128 (29.3)	103 (26.4)	
> 0	271 (79.7)	262 (68.4)	309 (70.7)	287 (73.6)	
SEALANTS					< 0.01^#^
= 0	334 (98.2)	367 (95.8)	430 (98.4)	267 (68.5)	
> 0	6 (1.8)	16 (4.2)	7 (1.6)	123 (31.5)	

^a^ Otherwise specified; °Kruskal-Wallis non parametric test; ^#^ Pearson’s Chi-square test

**Fig. 2:**
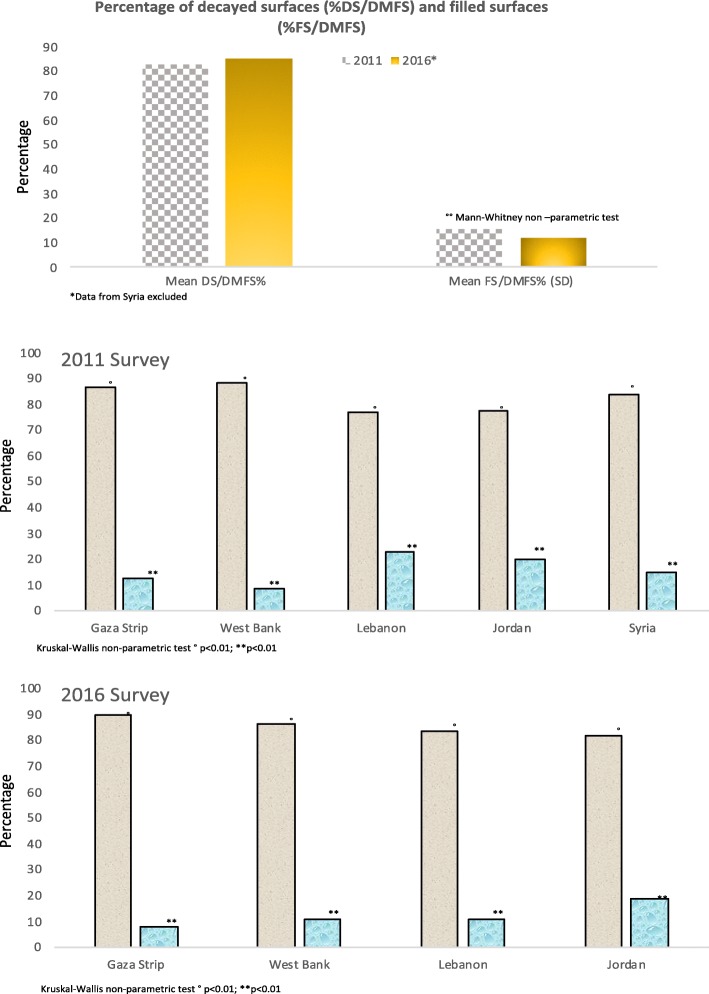
Percentage of decayed surfaces (%DS/DMFS) and filled surfaces (%FS/DMFS) among children with at least one decayed, missing, or filled permanent tooth surface (DMFS> 0), Overall, and by field and by gender in the two Surveys (2011 and 2016)

There were no differences in the DS/DMFS% between the two surveys (*p* = 0.13) while a difference in the FS/DMFS% (15.4% vs 11.6%, *p* < 0.01) (Fig. 2) was observed. A statistically significant difference was found among fields both for DS/DMFS% and for FS/DMFS% (*p* < 0.01 for all the comparisons). When stratified by gender no differences were found in the 2011 survey while results from 2016 showed that females had a lower value than male for DS/DMFS% (84.0% vs 86.9%, *p* = 0.01) and a higher value for FS/DMFS% (13.2% vs 9.9%, *p* < 0.01).

Among the most caries-exposed individuals (SiC index), the overall mean DMFS score was 9.2 in 2011 and 9.9 in 2016 (*p* < 0.01) (Table 4).

**Table 4:** SIC index overall, by field and by gender. The UNRWA oral health surveys 2011–2016

	Survey 2011				Survey 2016°				*p*-value
	N	Mean	SD	*p*-value	N	Mean	SD	*p*-value	< 0.01
Overall	906	9.2	4.73		424	9.9	4.41		
Field									
Gaza Strip	131	7.2	2.8	< 0.01°	116	10.2	4.4	0.43°	
West Bank	279	9.8	5.1		119	10.3	4.9		
Lebanon	147	10.3	5.7		118	10.0	4.5		
Jordan	153	8.5	4.5		71	9.1	3.3		
Syria	196	9.5	4.3						
Gender									
Male	452	9.2	4.6	0.58°	206	9.8	4.0	0.76°	
Female	454	9.2	4.9		218	10.2	4.8		

° data from Syria 2016 excluded

When analyzing data by field, a statistical difference of the DMFS SiC index was found in 2011 (*p* < 0.01), with the lowest in Gaza Strip (mean 7.2) and the highest in Lebanon (mean 10.3); while in 2016 no statistical difference was found, with the lowest in Jordan (mean 9.1) and the highest in West Bank (mean 10.3). No differences were observed between genders in both the 2011 and 2016 surveys.

Gingival health (bleeding on probing) also showed statistical differences among the fields. In comparison of 2011 and 2016 surveys, in general, an improvement was observed in Lebanon (*p* < 0.001), Gaza Strip (*p* < 0.001), West Bank (*p* < 0.01), and Jordan (p < 0.01) (data not shown in table).

### Oral health habits and dental attendance

**Fig. 3:**
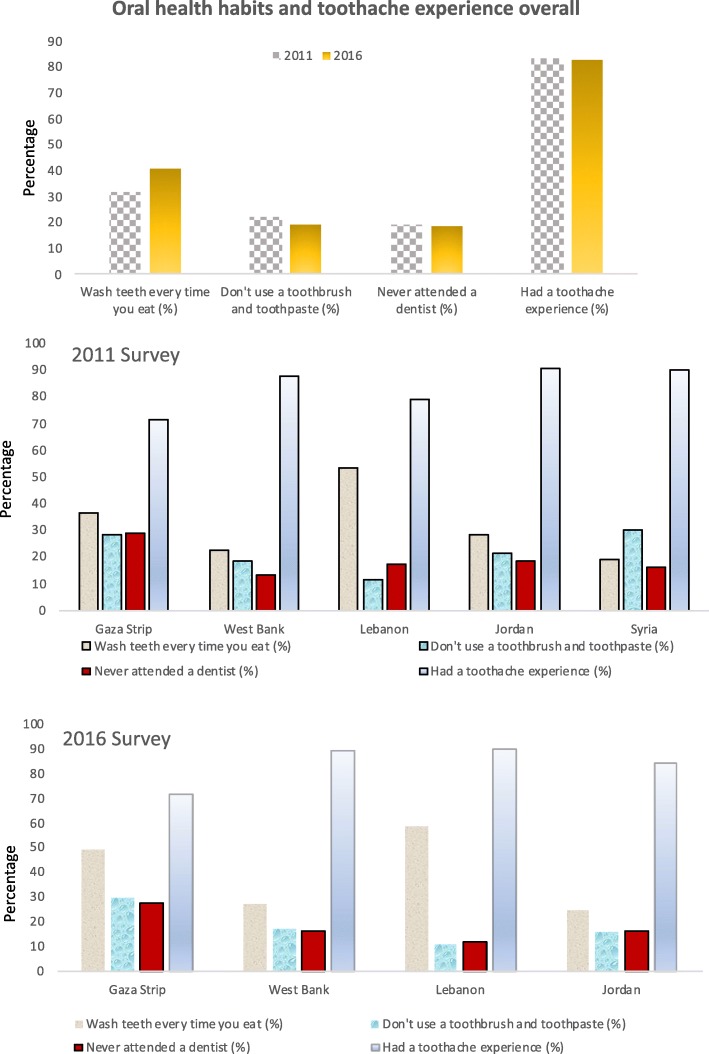
Percentage of schoolchildren with certain oral health habits and toothache experience overall and by fields in the two Surveys (2011 and 2016)

As shown in Fig. 3, in 2011, overall only 31.7% of students refer to cleaning teeth after every time they eat, while this percentage increases to 40.7% in 2016 (*p* < 0.01). Brushing frequency (Wash teeth every time you eat) was statistically significant different among the five fields with the highest frequency reported in Lebanon (53.2%) respect to the percentage reported in Syria (19.1%). In 2011, overall, 18.9% of students reported that he/she never attended a dentist, whilst 83.5% experienced a toothache in the past. These percentages remained similar in 2016 (18.3 and 83.1%, respectively) and these comparisons were not statistically significant (*p* = 0.67 and 0.72, respectively).

Dentist attendance and dental pain were associated with the prevalence of dental caries (DMFS> 0) in both surveys. Specific other confounders that were statistically significant in multivariate models included: soft drink consumption during meal, toothbrush habits, father tooth brushing and parents who attended a dentist in 2011 and soft drink consumption between meals and gender in 2016. (Tables 5 and 6).

**Table 5:** Logistic regression multivariate model for caries on permanent dentition (Survey 2011)

	OR [95% CI]	*p*-value
Soft drinks (during meals)		
Yes	1.00	
No	0.80 (0.67–0.95)	0.01
Toothbrushing habit		
Yes	1.00	
No	1.29 (1.07–1.56)	< 0.01
Father use of toothbrush and toothpaste		
Yes	1.00	
No	0.81 (0.66–0.99)	0.04
Dental attendance		
Yes	1.00	
No	0.70 (0.56–0.88)	< 0.01
Dental pain experience		
Yes	1.00	
No	0.46 (0.38–0.58)	< 0.01
Parents Dental attendance		
Both parents attend	1.00	
One parent attends	0.86 (0.70–1.05)	0.14
None attends	0.72 (0.54–0.95)	0.02

**Table 6:** Logistic regression multivariate model for caries on permanent dentition [Survey 2016 (data from Syria excluded)]

	OR [95% CI]	*p*-value
Gender		
Female	1.00	< 0.01
Male	0.71 (0.56–0.89)	
Soft drink between meals		
Yes	1.00	
No	0.75 (0.58–0.98)	0.03
Dental attendance		
Yes	1.00	
No	0.62 (0.47–0.84)	< 0.01
Dental pain experience		
Yes	1.00	
No	0.60 (0.45–0.812)	< 0.01

## Discussion

The two surveys provide reliable information on dental caries status and associated factors in representative samples of students enrolled in the UNRWA schools [7]. This information will serve the UNRWA Department of Health in planning and development of oral health promotion interventions.

The results indicate that in 2011, 73.1% of students across the five fields had caries experience in their permanent teeth (DMFT/S > 0) vs 72.8% in 2016 (*p* = 0.83). Both in 2011 and in 2016, the prevalence of caries experience was related to the field of residence.

In 2011, 68% of students had untreated tooth caries lesions (DT/S > 0) vs 69.4% in 2016 (*p* = 0.35). In the last decade, UNRWA has dedicated great efforts in improving the Oral Health Program with the aim of reducing the burden of the oral diseases [6, 8]. Despite this, the prevalence of caries experience and untreated tooth decay is high in all fields and no positive trend was observed between 2011 and 2016 concerning the main oral health indices. In particular, untreated caries lesions might affect seriously the students’ general health, nutrition, growth and body weight because of pain and discomfort, which could lead to acute and chronic infections and altered eating and sleeping habits [19, 20].

Moreover, caries data observed in both surveys are higher respect to those reported in surrounding countries [21, 22]; while the prevalence of dental caries of children of Middle East countries is reported moderate [23] it is necessary to take into account that the life conditions are quite different in each country and while the data derived from the two surveys are similar in Oman [24], caries prevalence was impressive higher in the two surveys respect to Jordan or Kuwait [21, 22].

Both surveys were found to have some limitations for the interpretation of the results. In general, a cross-sectional study measures cause and effect at the same point in time, therefore introducing the problem of temporal ambiguity and an inability to establish causal relationships. Among the limitations of this analysis, methodological difficulties in organizing and managing the data collection across five different fields are acknowledged. Additionally, the political instability which is typical for some of the regions, especially in Gaza Strip and Syria, influenced the precise definition of representative sample sizes and the actual data collection that resulted in delays.

Also, we must acknowledge that the questionnaire data source (12-year-old students self-reporting personal information under supervision) may be not reliable enough, specifically in relation to socioeconomic data.

## Conclusions

The 2011 and 2016 surveys have identified behavioral determinants for dental caries, particularly dietary habits such as soft drinks consumption, therefore confirming the crucial cariogenic role of added sugars [5, 25]. These results strongly suggest for the need of a large-scale integrated preventive approach toward oral health and the emerging growth of Non-Communicable Disease (NCD), in line with WHO recommendations [26, 27]. In fact, NCD and oral pathologies share many of the same risk factors such as excessive consumption of sugar or alcohol and widespread use of tobacco: these are preventable risk factors that are related to lifestyles. For this reason, NCD and oral diseases should be addressed together [27–30].

In particular, WHO recommends the WHO-PEN strategy (WHO Package of Essential Non-communicable Disease Interventions for Primary Health Care in Low-Resource Settings) [27] to address the burden of diseases that share many risk factors, by packaging together a number of health interventions. UNRWA works in partnership with WHO and other UN agencies, so the management and the approach for the UNRWA Health Program recommended by WHO is therefore strongly encouraged and eased.

UNRWA, in order to address the growing burden of NCD, has started to focus on the prevention and control of the NCD through an articulated multilevel strategy [6, 9]. In particular, the UNRWA School Health Strategy [8] – that reflects the FRESH global framework [31] for school health - aims to tackle health risk behaviors. In the case of oral disease the effort to promote at school adequate attention to hygiene, nutrition and diet will reinforce and benefit other important aspects of general health.

## Data Availability

The English version of the administered questionnaire and datasets generated during and analyzed during the current study are available from the corresponding author on reasonable request.

## Abbreviations

COI: Cooperazione Odontoiatrica Internazionale
CPI: Community Periodontal Index
DMFS: Decayed, missing, filled surfaces
DMFT: Decayed, missing, filled teeth
DT/S: Decayed teeth/surfaces (DT/S)
FOHSOs: Palestine Field Oral Health Services Officers
FS: Filled Surfaces
FT: Filled Teeth
MS: Missing Surfaces
MT: Missing Teeth
NGO: Non-Governmental Organization
SD: Standard Deviation
SIC: Significant Caries index
UNRWA: United Nation Relief and Work Agency for Palestine refugees in the Near East
WHO: World Health Organization
